# William Baly

**Published:** 1861-04

**Authors:** 


### In Memoriam.

### (WILLIAM BALY.)

In offering a few observations in reference to one who has so recently been amongst us, were we for a moment to permit personal feelings to influence the discharge of public duties, we should rest satisfied with recounting to the profession instances of the gentle nature, kind disposition, and great personal worth of him whose lamentable death we, in common with all who had the privilege of his acquaintance, mourn. To those who knew Dr. Baly, his characteristics are familiar. To those who may be led to inquire by what means one who, having entered the medical profession without the external advantages generally regarded as essential to success, attained to its highest position, and at an early age was followed to his grave by the great and gifted of the land, an impartial scrutiny of his life may not be without advantage. We have already* impressed on our readers, as a great fact, that success is the result of merit, and endeavoured to point out to those entering upon medical studies the necessity for a patient, painstaking spirit of inquiry, and steady earnestness in the pursuit of their profession. To the sceptical in reference to the sufficiency of intellectual and moral excellence for the attainment of the highest success, we again detail a career singularly illustrative of opinions anxiously enunciated as truths—right in principle and proved in practice.

William Baly was born at Lynn, in Norfolk, in 1814. In his early career there is little special interest. In common with the majority of English youth of the middle class, he received a sound preliminary education in the Grammar School of his native town. His father, while engaged in the active pursuit of provincial commerce, found time and opportunity for the cultivation of general literature, the fascinations and advantages of which he early impressed on his son. His mother, who united to a highly cultivated mind good sense and refined judgment, insensibly exercised that guiding influence to which, more than to all other causes combined, is attributable the success of the greatest men. Both parents contributed to their son's future proud position; from the one he inherited an active persevering honesty of character which, when

* See last number—Memoriam (Dr. Todd).

associated with genius, ever offers an irresistible combination; from the other he derived a due appreciation of those higher studies and duties which subsequently guided and controlled his ambition. Impressed with the practical reality of life, the character of his home education influenced his conduct throughout the future, and imparted to his actions an apparent timidity which, to those who knew him, was nothing more than the struggle of prudence with ambition. At an early age young Baly expressed a wish to enter the medical profession; which, to a mind so constituted, must ever offer special attractions. He was accordingly apprenticed to Mr. Ingle (now Dr. Ingle), of Emsworth, a general practitioner of high professional and personal repute. From this gentleman he derived that rudimentary knowledge which subsequently bore such splendid fruit. The apprentice soon mastered the details of duty presented by country general practice. An experimental and practical acquaintance with pharmacy as a means to an end, a capability of recognising disease in its familiar forms, a certain degree of intimacy with the general run of surgical cases, acquired under the kindly guidance of Dr. Ingle, prepared his mind for entering on that large field of observation and practice to which too many students are annually introduced without previous questioning of their fitness or capacity. In 1831 Mr. Baly entered University College as a pupil. Diligent in his attendance on lectures, constantly present in the anatomical school, he laid a sure and sound foundation for future practical success by acquiring a thorough acquaintance with the Organism, and a complete theoretical knowledge of the laws which experimental philosophy demonstrates as influencing the operations of life. In the following year he entered at St. Bartholomew's Hospital. Here, to a mind prepared for observation, what to many were inexplicable difficulties, proved great opportunities. In the practical observation of disease he attained the consummation of his studies; in its diagnosis he tested the fidelity of his impressions; in its treatment he proved the truth of his inferences. Earnest and energetic in his pursuit of the bedside investigation of disease, he soon attracted the notice and gained the approbation of the clinical physicians, Drs. Latham and Burrows. To the former much of his subsequent success is due. From both he then and throughout life experienced many proofs of confidence and esteem. So impressed was each of these gifted and highly distinguished men with the capacity and character of their clinical pupil that they severally counselled him to settle in the metropolis, and to venture on the life of a London physician. Their advice determined his career. In 1834, having passed the College of Surgeons and the Apothecaries' Hall, he repaired to Paris, where he devoted the following winter to professional studies. In the spring of the ensuing year he proceeded to Heidelberg, and thence to Berlin, where, in 1836, he graduated as Doctor of Medicine. How fully Dr. Baly availed himself of the advantages of the several continental schools may be inferred from his intimacy with the opinions, writings, and practice of their most distinguished professors, with many of whom he had even then the privilege of friendly personal communication.

On his return to England, Dr. Baly commenced practice. By this the reader must understand that, virtually unknown in London, without other introductions than those which his professional exertions had gained, without friends except those which his own high personal character had secured: and with limited pecuniary means, he fixed his residence in Vigo-street, and by placing his name on its door, informed the public that a stranger anxious for their support had come among them. The first years of professional life are trying to every man. To the youth, enterprising and ambitious, who stands alone in the vast arena of metropolitan competition, each day is fruitful in stern lessons of perseverance and self-denial. Before him is the proof of success; around him are difficulties which appear to be insurmountable; pressing necessities of the present must be provided for, while great expectations for the future are not relinquished. It requires a stout heart, high hope, self-reliant energy, and a well-disciplined mind to divest disappointment of its bitterness, and to bear up against the sickness of heart which results from hope deferred. Success is certain sooner or later to those so endowed. Small intelligences growing faint and weary on the way, and unequal to the strife, rest satisfied with respectable mediocrity. They are not, therefore, the less happy in their career. Success beyond their attainment is seldom within their capacity. Men of ability and energy ever triumph. They do surmount opposition. They create, if they cannot command circumstances, and prove equal to the opportunity. Dr. Baly was an example of this truth. Within a year after his return to England he was actively employed as medical officer to St. Pancras Infirmary, to which post he was elected solely on account of the high character he had established during his pupilage, and the deserved reputation he had acquired while abroad as a student. At the same time he was induced to undertake the translation of Müller's *Physiology*, a work which will always live as a monument of its author's great genius and of Dr. Baly's untiring industry. The opinion entertained by the profession of the manner in which this important trust was discharged need not be recapitulated. While the text of the book proved Dr. Baly to be an accomplished scholar, its notes evinced him to be a painstaking experimentalist and an expert and original investigator. This arduous and responsible work was undertaken not from any special predilection for the science of which it treated, but rather as a means to an end—a present source of income, consistent with a course of study, indirectly though not immediately available for the practical purposes of his profession. On the appearance of this work Dr. Baly took deserved rank amongst men of science. His teachers and friends felt that their opinions were well founded, and their expectations just. In his new position Dr. Baly evinced the same steadfastness of purpose, sobriety of thought, and forcible simplicity of judgment, which having secured for the student friendship, won for the practitioner respect. It will be seen that the impressions then made, and subsequently fully realized, were not without their practical results. The youth of the profession should lay these facts to heart. They are the true lessons of success in life.

In 1840, epidemical disease prevailed in London. In Millbank Penitentiary, more especially, dysentery was very prevalent. On Dr. Latham's recommendation Dr. Baly was directed to visit and report on its sanitary condition. The manner in which he discharged this duty was eminently satisfactory to those who had appointed him. A vacancy occurred in the following year, and Dr. Baly was at once elected as physician to that establishment, which position he continued to hold until his death. To a young man of Dr. Baly's ability and circumstances such an appointment was invaluable. Anxieties in reference to pecuniary matters were at an end, while ample opportunity for the observation of disease was afforded. How fully he availed himself of the latter, and how amply he repaid the confidence of his teachers, the medical profession well know. They, however, are not alone in the benefits which resulted from this appointment. To Dr. Baly is due that revolution in the hygienic management of prisons which late years have witnessed. In constant communication with successive Home-secretaries, Inspectors, and Governors, he impressed on the authorities the truth now generally acknowledged, that the physical and moral condition of man are inseparably united. Prisons have ceased to be pest houses. Punishment now acts on the mind equally with, or we might add, rather than on the body; and, while the law is vindicated, humanity is no longer outraged. Dr. Baly's elaborate paper on "Diseases of Prisons," in the twenty-eighth volume of the *Medico-Chirurgical Transactions,* his Gulstonian Lectures on Dysentery, published in 1847, manifest a minute and accurate observation of their several subjects sufficient to explain the confidence which those in authority reposed on his experience and judgment.

In 1841 Dr. Baly was appointed Lecturer on Forensic Medicine at St. Bartholomew's Hospital. This lectureship he held for fourteen years, discharging its duties with credit to himself and advantage to his classes; nor did he resign it, until he was called to the higher and more responsible office of assistant-physician to the same great institution, and in conjunction with Dr. Burrows, his former teacher, to the lectureship on Medicine in its distinguished school. To the former of these positions he was elected in 1854, to the latter in 1855. In the interval between his appointment to Millbank and to the physicianship of St. Bartholomew's, Dr. Baly had grown in favour with the profession and the public. At the desire of the College of Physicians (of which body, in 1846, Dr. Baly had been admitted a fellow), he undertook to report on cholera. To those who have read and studied his volume a recommendation is needless. To those who have not done so, we unhesitatingly declare, that if Dr. Baly's reputation had rested on it alone, he had written enough to render himself eminent.

In 1847 he was elected a fellow of the Royal Society, a distinction reserved for men who can found special claims on original research in science.

From Dr. Baly's appointment to St. Bartholomew's Hospital may be dated his prominence amongst his professional brethren. He was soon most highly esteemed by his colleagues, and very popular with the students. His teaching was highly practical, he was readily accessible to every one seeking information, and always anxious to impart knowledge. The patient gave confidence to ability which each day practically demonstrated; the pupil respected opinions never hastily formed, and generally verified. The principle of his life stood declared at each step of his progress, it was this—always to enter on a duty with firm resolve to approximate to its perfection, if he could not wholly achieve it. That which he undertook to do, he did well. Without the startling attributes of genius by which men have risen from the ranks of every-day life to exalted positions, he possessed those qualities which, in practical life, must ever achieve a certain success—clear intelligence, ambition regulated by principle, and a heart whose affections and impulses were influenced by religion. His was not the nature to force its way into pre-eminence, but having been placed in the highest position, the duties and responsibilities which it entailed entered into the routine of his daily practice as part of his habitual vocation. Such a disposition required but opportunity to ensure success in a profession where the heart and head co-operate for a common purpose—the relief of suffering humanity.

The deserved reputation Dr. Baly had acquired in the opinion of the authorities with whom, from his official position, he was brought into relation, caused him to be consulted by the Crown on most of the grave jurisprudential questions which have arisen of late years. We know that to his report is mainly, if not entirely due, the remission of sentence in a remarkable case which a year or two since engaged the public mind. His official character stood as much above reproach as his professional capability above question. It is not surprising that when opportunity offered to those who had proved his capacity and known his excellence, to select one for the highest position open to a physician, their choice should have proved a corroboration of the confidence which Dr. Baly had fairly earned, and justly enjoyed. In 1859 it became requisite to select a physician who might at first share with Sir James Clark, and then hold alone, the office of regular attendant on the Queen and Royal Family. To this distinguished duty Dr. Baly was called. The unanimous voice of the profession approved the wisdom of the selection.

During the short time Dr. Baly held this high office, he received many proofs of confidence from the Queen and Prince Consort; not the least of which was his nomination to a seat in the Medical Council as one of the representatives of the Crown, in the place of Sir James Clark. In the meridian of life, and the full vigour of intellect, he seemed destined to a long and prosperous career; but Providence ordered it otherwise. All are familiar with the lamentable accident which resulted in his death; but all do not know that though the cause was unforeseen, the victim was not unprepared. Men who live as Dr. Baly lived need not fear to die. His mind was schooled in the truths of religion, which regulated his thoughts and governed his life.

The immediate circumstances of Dr. Baly's demise cannot too deeply or too long rest in the minds of those whose daily occupations show that death and destruction are around each path, while danger floats in the very air they breathe. On Monday afternoon, the 28th of January, Dr. Baly attended as usual his hospital duties. In health and spirits he conversed with his class. He mentioned that he had received a telegram summoning him to Guildford. He drove to the Waterloo station, and in his haste left both card-case and note-book in his carriage. He proceeded by the train which leaves London at ten minutes past five. Some short distance beyond the Wimbledon station the tender and five of the carriages of the train were jerked off the rails, and rolled down an adjoining bank. The carriage in which Dr. Baly was sitting, by the force of its oscillation, jerked him through its opening. He fell on the line of rail. The carriage immediately following turned over upon him, and crushed him to the earth. It is a sad consolation to know that from the nature of the injuries death must have been instantaneous. His temple was beaten in, his side pressed against his spine, his mouth was filled with sand, and his features were so disfigured, that when drawn from beneath the carriage his mangled remains were incapable of identification. His body was removed to a small inn at Malden, where it remained unrecognised. As he did not return during the night, his sister, on the following morning, sent word to the hospital that he had been detained in the country, and could not deliver his usual lecture. The gentleman who had sent for him, hearing of the accident, and alarmed at his non-arrival, went to town. On the following morning a stranger called at the hospital, and requested to see the medical officers. The calamity was then made clear. It is unnecessary to detail the long and searching investigation which the duty and affection of his executor and friends caused to be instituted on the coroner's inquest; it is sufficient to add that the occasion of the accident was so involved in scientific subtlety, that the inquiry terminated in the usual anomalous decision characteristic of such tribunals. The fact of Dr. Baly's death remains, while the origin of the accident is undetermined. On this fact it is our sad office to comment. His life afforded a cheering and encouraging example, his death has added a solemn and impressive moral. It speaks of the uncertainty of life, the vanity of human joys, and the equality of the grave, in language that cannot be misunderstood.

In our last number we offered to our medical youth the encouraging assurance that merit and perseverance seldom fail to create friends, and conquer difficulties. We then traced the career of Dr. Todd. Alas! that so soon we should be called upon again to record labours crowned with equal success, and a career prematurely and sadly terminated. Yet so it is. If men were empowered to regulate their own future, who is bold enough to affirm that he could improve his condition? The mysterious providence of God controls the movements of each; and is not the less merciful, because we grieve at its manifestation, and not the less loving, because its operation is beyond our finite comprehension.

